# Delayed Posthypoxic Leukoencephalopathy in a Catatonic-Appearing Patient: A Case Report

**DOI:** 10.1155/crps/9978149

**Published:** 2025-08-29

**Authors:** Meredith Bentley, Jordan Gaal, Janice Hostetter, Suzanne Holroyd, John Pickstone, Kelly Melvin

**Affiliations:** Department of Psychiatry and Behavioral Medicine, Marshall University Joan C. Edwards School of Medicine, Huntington, West Virginia, USA

## Abstract

Delayed posthypoxic leukoencephalopathy (DPHL) is a rare diagnosis that may present similarly to other more common neurological conditions, such as catatonia. While often seen with carbon–monoxide poisoning, it can also be due to anoxia due to other causes, such as drug overdose or cardiac arrest. Due to the delayed nature of its symptoms and overlap with other conditions, it can be initially misdiagnosed. We present a 50-year-old female patient with a history of depression who was found unresponsive, hypoxic, and febrile at her home for an unknown amount of time. The initial concern was for sepsis. Initial computed tomography (CT) of the head and magnetic resonance imaging (MRI) of the brain were normal. The patient had rhabdomyolysis with secondary renal failure, shock liver, and acute pancreatitis. Once medically stabilized, her cognition returned to a normal baseline. However, 10 days into her hospitalization, her mental state deteriorated, displaying symptoms of mutism, stupor, staring, decreased oral intake, and perseveration. Catatonia, secondary to a major depressive episode, was suspected. Lorazepam was titrated upward without result. Lack of response to lorazepam prompted a repeat brain MRI, revealing diffuse white matter changes in the frontal, temporal, parietal, and occipital lobes of both hemispheres. A diagnosis of DPHL was made. She was then started on carbidopa/levodopa 25/100 mg with improvement and was discharged to a rehabilitation facility.

## 1. Introduction

Delayed posthypoxic leukoencephalopathy (DPHL) is a syndrome of cerebral demyelination following hypoxic brain injury [1]. After the initial hypoxic event, there is a nonsustained return to a baseline function which lasts days or weeks but is ultimately characterized by a rapid decline in neurobehavioral function. For some patients, the clinical presentation is primarily parkinsonian, but others display symptoms consistent with akinetic mutism [1]. Establishing the diagnosis can be a challenge due to the delayed nature of symptom onset and limited laboratory and neuroimaging options early in the course of illness. Magnetic resonance imaging (MRI) is often normal upon hospital admission but reflects leukoencephalopathic changes later in the disease process [2]. Either subtype of DPHL has high symptom overlap with other more commonly encountered clinical conditions, and diagnosis requires a high index of suspicion [3].

Catatonia is a neuropsychiatric syndrome characterized by the presence of cognitive, motor, speech, and behavioral symptomatology. Mutism, staring, posturing, and catalepsy are some of the most well-known signs, but clinical presentation varies dramatically [4]. Various scales, such as the Bush–Francis Catatonia Rating Scale, help gauge severity and assist with diagnosis [4]. Treatment is most often with a benzodiazepine [5]. Other conditions, such as akinetic mutism, status epilepticus, locked-in syndrome, and stroke may mimic catatonia. In addition, it is important to rule out malingering, factitious disorder, sluggish obsessive-compulsive disorder, and other neuropsychiatric conditions when considering a diagnosis of catatonia. Differentiating catatonia from other conditions requires careful history, examination, and selected laboratory or neuroimaging findings, including a lumbar puncture. We present a case of DPHL, presenting with akinetic mutism originally misdiagnosed as catatonia, showing the difficulty of diagnosis of DPHL and differentiating it from other conditions.

## 2. Case Presentation

**Figure 1:**
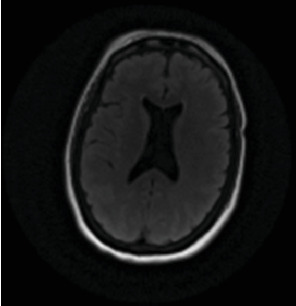
MRI performed upon admission revealed no abnormalities.

**Figure 2:**
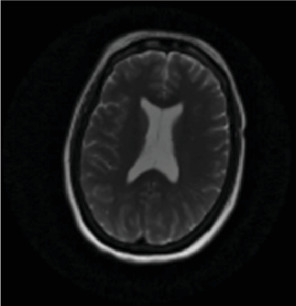
Additional image from admission using T2 imaging.

**Figure 3:**
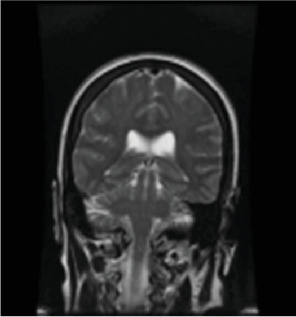
Coronal FSE T2 image from admission.

**Figure 4:**
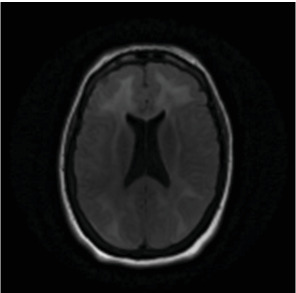
Follow-up MRI showing widespread subcortical white matter hyperintensity.

**Figure 5:**
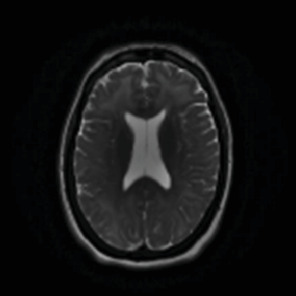
Additional follow-up image using T2 imaging. Impression: New extensive abnormal increased T2/FLAIR signal in both cerebral hemispheres with somewhat symmetric distribution. Findings are nonspecific, although the distribution suggests toxic or metabolic etiology.

**Figure 6:**
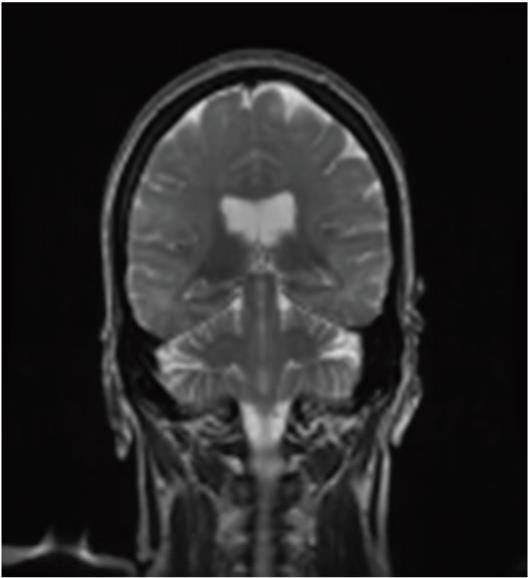
Coronal FSE T2 image on repeat MRI revealing extensive subcortical white matter hyperintensity in the midline parietal cortex, cerebellum, and brainstem.

A 50-year-old female was found unresponsive in her home for an unknown duration and was brought to the hospital while she was febrile (40.22°C). She had a history of anxiety and depression, treated with escitalopram 20 (mg) once daily, buspirone 15 mg twice daily and quetiapine 25 mg at bedtime. She had no known hospitalization or history of psychosis. As summarized in Table 1, the initial concern was for sepsis, with an initial white blood cell count being 13.8 1000/mm^3^ (*H*), total neutrophils being 12.806 1000/mm^3^ (*H*), and segmented neutrophils being 92.8% (*H*), but urine and blood cultures came back negative. Computed tomography (CT) of the head and MRI of the brain were also initially normal (Figure 1, 2, 3). Lumbar puncture showed normal protein and glucose, one white blood cell and 46 red blood cells. The one white blood cell was within the normal range of 0–5 cells, and the increased number of red blood cells was presumed to be from a traumatic tap. Thus, ruling out any infectious cause. The patient was admitted to the intensive care unit (ICU). The patient was in renal failure (potassium level of 6 mmol/L; blood urea nitrogen (BUN) of 30 mg/dL; creatinine of 3.6 mg/dL) and had rhabdomyolysis with elevated creatinine phosphokinase (total Creatinine Kinase of 2958). Dialysis was required and initially the patient required continuous renal replacement therapy and eventually transitioning to hemodialysis. Liver function tests were elevated on admission (aspartate aminotransferase (AST) 1547 international units per liter; alanine aminotransferase (ALT) 328 international units per liter; alkaline phosphatase 182 IU/L) with a diagnosis of shock liver, and acute pancreatitis was diagnosed with elevated amylase and lipase (Table 1). On day three, renal function began to stabilize, thus no longer necessitating dialysis. Initially her mental status improved to be at a normal baseline (Table 1). On day six, the patient was transferred out of the ICU. As shown in Table 1, she was treated with a 10-day regime of clindamycin for superficial thrombophlebitis and again required dialysis on day 11 of admission. On day 19, mental state acutely deteriorated, showing symptoms of mutism, stupor, staring, decreased oral intake, perseveration, and inability to follow commands. Neurology was consulted (Table 1). A repeat CT showed no acute processes and an electroencephalogram (EEG) showed generalized slowing with superimposed frontal intermittent rhythmic delta activity (FIRDA) suggestive of generalized encephalopathy. Psychiatry was consulted initially for delirium. Escitalopram and buspirone were held, and quetiapine was scheduled at 25 mg by mouth at bedtime to help with sleep, and quetiapine 25 mg was ordered as needed for any behavioral issues or agitation. Her mental status did not improve, and she developed cogwheel rigidity in her right wrist. Quetiapine was thus discontinued, and lorazepam was started for possible diagnosis of catatonia due to a major depressive episode (Table 1). Lorazepam was titrated to 1 mg three times daily for 6 days, but without improvement, it was subsequently tapered and discontinued. Lack of response to lorazepam caused doubt that this was in fact catatonia, prompted repeat MRI of the brain revealing increased signal on T2-weighted fluid-attenuated inversion recovery (T2/FLAIR) in frontal, temporal, parietal, and occipital lobes in both hemispheres (Figure 4, 5, 6; Table 1). DPHL was diagnosed (Table 1). Carbidopa/levodopa 25/100 milligrams was started for encephalopathy per neurology. A repeat lumbar puncture was obtained with fluid analysis that was unremarkable, showing zero red or white blood cells, normal glucose, and a protein level elevated only slightly above the upper limit of normal. The patient improved significantly and was ultimately discharged to a rehabilitation facility, to follow-up with nephrology and neurology 1 month afterward.

## 3. Discussion

DPHL has been called many things in the literature, including delayed postanoxic leukoencephalopathy and delayed neurological sequela of carbon monoxide poisoning or other anoxic events [6]. Common causes of DPHL include carbon monoxide intoxication and respiratory failure from drug overdose and myocardial infarction [6]. Classically, patients will recover fully from their initial coma only to then either days or weeks later, present with new-onset neurological symptoms, such as dissociation, amnesia, hyperreflexia, parkinsonism, akinetic mutism, or psychosis [6]. Common differential diagnoses for DPHL include multiple sclerosis, acute disseminated encephalomyelitis, allergic encephalomyelitis, progressive multifocal leukoencephalopathy, radiation induced delayed encephalopathy, chemotherapy-induced neurotoxicity, and several other more rare forms of leukodystrophy [3]. The level of hypoxia needed for an insult to cause DPHL is also unclear, as is the role of arylsulfatase—A deficiency or pseudodeficiency in the pathophysiology of DPHL [3]. Arylsulfatases catalyze the hydrolysis of sulfate ester bonds in glycosaminoglycans, sulfate esters, small aromatic molecules and sulfolipids. Arylsulfatase A specifically is found in lysosomes [7].

Upon examination of the histopathological characteristics of DPHL, compared to that of acute-hypoxic ischemic injury, DPHL is characterized by widespread symmetrical alterations predominantly in the white matter regions, whereas acute ischemia is associated with localized changes in the gray matter structures [8]. It has been suggested that the lucid interval between the hypoxic event and the noted decline in brain function is related to the mean replacement half-life for myelin related proteins and lipids of 19 days [9]. A potential theory as to why the damage is limited to the white matter is that oligodendrocytes, some of which are responsible for the production of myelin, are uniquely vulnerable to damage from glutamate-induced excitotoxicity. This hypoxic event leads to depletion of ATP, thus leading to release of glutamate [6].

Catatonia classically presents as marked kinetic impairment and can be seen secondary to autism spectrum disorder, bipolar disorders, psychotic disorders or unipolar major depression, as well as general medical disorders. The most common presenting kinetic signs are immobility, rigidity, mutism, posturing, excessive motor activity, stupor, negativism, staring, and echolalia. There are three types of catatonia, based upon the kind and type of movement disturbance; retarded, excited, and malignant [5]. Overlap of catatonia and many other neurological disorders can be difficult, as it was in this case.

## 4. Conclusion

In the case of this patient, it was only apparent that there was a misdiagnosis of catatonia once the lack of improvement of symptoms was noted after lorazepam administration [5]. DPHL was then able to be diagnosed after imaging showed the classical presentation of symmetrical and restricted white matter changes [8]. DPHL should be considered for patients with catatonia-like symptoms who do not respond to a trial of lorazepam and whose presentation involves a hypoxic event with return to baseline cognition and then followed by neurobehavioral decline.

## Figures and Tables

**Table 1:** Timeline of case evolution and key events.

Timeline of case evolution and key events	
Day 1	Diagnoses made for sepsis, renal failure, rhabdomyolysis, and shock liver. CT and MRI were normal. Dialysis started.
Day 3	Kidney function normalized, dialysis discontinued, would resume PRN only
	Mental status improved to baseline
Day 6	Transferred out of the ICU
Day 11	Clindamycin started for superficial thrombophlebitis. Dialysis required.
Day 19	Mental status deteriorated, psychiatry consulted for suspected delirium. Seroquel was started.
Days 21–26	Trial of lorazepam for suspected catatonia
Day 27	Repeat MRI revealing DPHL

## Data Availability

The data that support the findings of this study are available from the corresponding author upon reasonable request.
